# Chronic conditions, multimorbidity, and quality of life among patients attending monk healers and primary care clinics in Thailand

**DOI:** 10.1186/s12955-021-01707-x

**Published:** 2021-02-23

**Authors:** Supa Pengpid, Karl Peltzer

**Affiliations:** 1 ASEAN Institute for Health Development, Mahidol University, Salaya, Phutthamonthon, Nakhon Pathom, Thailand; 2 Department of Research Administration and Development, University of Limpopo, Turfloop, Mankweng, South Africa; 3 Department of Psychology, University of the Free State, Bloemfontein, South Africa

**Keywords:** Chronic conditions, Multimorbidity, Quality of life, Monk healer, Public primary care, Thailand

## Abstract

**Background:**

The study aimed to assess chronic diseases, multimorbidity, and QoL among patients attending two different treatment settings in Thailand.

**Methods:**

In all, 1409 attendees of three monk healer or three health centres were assessed with self-reported measures on chronic conditions and Quality of Life (QoL).

**Results:**

Results indicate that the most common chronic conditions were common mental disorder (25.2%), followed by hypertension (22.8%), high blood cholesterol (18.0%), fatigue disorder (14.4%), diabetes (14.0%), migraine headaches (13.7%), sleeping problem (12.2%), and ulcer (11.0%). In all, 40.6% had multimorbidity (two or more chronic conditions) (42.4% in the monk healer and 38.9% in the primary care setting). In ANCOVA analysis, adjusted for sex, age, employment status, marital status, education, economic status, comorbidity, and health care setting, the poorest overall QoL was found among clients with common mental disorders (58.5 mean score), followed by emphysema or asthma (60.2), sleeping problem (61.5), migraine headaches (62.7), fatigue disorder (63.3), substance use disorder (63.6) and ulcer (64.3). The overall QoL was poorer among monk healer clients (66.5) than primary care patients (68.8). In adjusted logistical regression analysis, being a monk healer attendee, older age (55–93 years), and high debt were positively, and being employed and better overall quality of life were negatively associated with multimorbidity, overall, for the monk healer and primary care setting. In adjusted linear regression analyses, primary health care attenders, older age, were employed and post-secondary education increased the odds of better overall QoL.

**Conclusion:**

Multimorbidity was higher among clients attending monk healers than those attending primary care facilities and QoL was poorer among clients seeking care from monk healers than those attending primary care. High multimorbidity was found and major chronic conditions were found to have poor QoL. Determinants of multimorbidity and QoL in two different treatment settings provide information to improve the management of chronic conditions.

## Background

Chronic conditions are common among patients in primary care settings. Multimorbidity (coexistence of two or more chronic conditions) has been found in Southeast Asian countries [1]. In Odisha state of India, 28.3% of patients (≥ 18 years) in primary care (30.7% in public care and 24.6% in private care) had multimorbidity [2], and in rural primary care in Kerala, India, the prevalence of multimorbidity was 16.2% (≥ 18 years) [3]. In a study among chronic disease primary care patients in four Greater Mekong countries, the majority (72.6%) had multimorbidity [1]. In Singapore, among adult patients with chronic diseases in public sector primary care clinics, 39.6% of those who had used traditional and complementary medicine (TCM) in the past year and 28.1% who had not used (TCM) had multimorbidity [4]. Several studies [4–7], including in Cambodia, Thailand, Singapore, USA, and Vietnam, showed that patients with multimorbidity are more likely to utilize traditional and complementary medicine (TCM) than patients without multimorbidity. In a further study, multimorbidity of chronic physical and mental illnesses was associated with higher TCM use [8]. In comparing the morbidity pattern of patients of traditional Chinese medicine practitioners with Chinese medical practitioners in Hong Kong population, showing that for chronic illnesses TCM was the preferred treatment [9]. Multimorbidity has been identified as a major challenge for health care systems, which are based on single disease-specific management [2]. Therefore, an understanding of the prevalence of multimorbidity in both a TCM and conventional primary health setting are vital in designing more effective primary care [2]. There is, to our knowledge, no information on multimorbidity in a traditional health practitioner (THP) setting. It is hypothesized that attendees of THP (monk healer) have a higher prevalence of multimorbidity than primary care attendees. Monk healers are Thai THP providing various types of treatments, including herbal medicine, physical therapy, and Buddhist practices, such as prayer [10, 11].

Chronic conditions persist for a long time and affect functional ability and quality of life negatively [12]. “Health-related quality of life (HRQoL) is a multi-dimensional concept that includes domains related to physical, mental, emotional, and social functioning” [13], and is commonly used to describe overall well-being and health [14] and general health needs [15]. Several studies [1, 12, 16, 17] show that multimorbidity in primary care patients is negatively associated with QoL. Among primary care patients in Southeast Asian countries, poorer QoL was identified in cancer, mental disorders, asthma, chronic obstructive pulmonary disease (COPD) and cardiovascular diseases (CVD) [18]. Among Chinese primary care patients, depression and osteoarthritis had the lowest QoL compared to many other chronic diseases [19]. In a multicountry study, arthritis, COPD and CVD had the lowest QoL scores [20]. We did not find a study describing the overall QoL among attendees of THP, however, in a study in Brazil, the overall QoL was 65.2 (range 0–100) among attendees of primary health care [21]. It is not clear if QoL among attendees differs by monk healer or primary care setting. It was hypothesised that together with a higher prevalence of multimorbidity in the monk healer compared to the primary care setting, QoL would be poorer among clients attending monk healers than those attending primary care.

As reviewed previously [1], factors associated with multimorbidity may include older age, female sex, lower socioeconomic status, and low QoL. Lower QoL among primary care patients, as previously reviewed [18], may be associated with multimorbidity, older age, being female, not married or cohabiting, lower education, rural residence and substance use. The study aimed to assess chronic diseases, multimorbidity, and QoL among patients attending monk healers and primary care health centres in Thailand. Study findings could help in identifying patient groups with differentially impaired QoL within multimorbidity and designing specific care plans.

## Methods

### Design and participants

Using a cross-sectional study design, 1409 adult attendees of two treatment settings (primary care and monk healer) were interviewed by a trained professional nurse consecutively from six sites in the eastern and central region of Thailand over a period of four months in 2018/2019. Study sites were purposefully selected with the inclusion criteria that they had at least five adult patients per day. The “Office of The Committee for Research Ethics (Social Sciences), Mahidol University (No.: 2017/055.1403)” approved the study, and written informed consent was provided by participants.

### Measures

*Sample characteristics* included marital status, highest educational level, gender, age, work, and economic status (extent of debt).

Chronic conditions. Clients were asked about 16 health care providers’ diagnosed chronic conditions, such as asthma, diabetes, emphysema, and hypertension (see Table 1).

**Table 1:** Sociodemographic characteristics and percentage of multimorbidity (≥ 2 chronic conditions)

Variable	Monk healer (n = 686)		Health center (n = 723)		Total (N = 1409)	
	Sample % (95% CI)	Multimorbidity % (95% CI)	Sample % (95% CI)	Multimorbidity % (95% CI)	Sample % (95% CI)	Multimorbidity % (95% CI)
Age group						
19–39	30.6 (27.0, 34.4)	43.0 (35.8, 50.5)	16.1 (13.4, 19.1)	16.7 (10.6, 25.2)	23.1 (20.8, 25.5)	33.2 (27.9, 39.0)
40–54	39.2 (35.3, 43.1)	42.1 (35.8, 48.6)	33.2 (29.7, 37.0)	33.7 (27.5, 40.4)	36.1 (33.4, 38.9)	38.1 (33.6, 42.7)
55–93	30.3 (26.7, 34.1)	59.6 (52.1, 66.8)	50.7 (46.8, 54.6)	59.0 (53.3, 64.3)	40.9 (38.1, 43.6)	59.2 (54.7, 63.5)
Sex						
Female	75.5 (71.9, 78.8)	49.3 (44.6, 54.0)	72.7 (69.1, 76.0)	41.5 (37.0, 46.2)	74.1 (71.5, 76.5)	45.4 (42.1, 48.7)
Male	24.5 (21.2, 28.5)	41.7 (33.6, 50.3)	27.3 (24.0, 30.9)	47.2 (39.6, 54.9)	25.9 (23.5, 28.5)	44.7 (39.1, 50.5)
Formal education						
Primary or less	38.5 (34.7, 42.5)	47.7 (41.0, 54.4)	64.6 (60.8, 68.3)	52.1 (47.1, 57.0)	52.3 (49.2, 54.8)	50.5 (46.5, 54.5)
Secondary	31.7 (28.0, 35.6)	43.1 (35.9, 50.6)	26.6 (23.2, 30.2)	23.6 (17.7, 30.8)	29.0 (26.5, 31.7)	33.7 (28.9, 39.0)
Post-secondary	29.8 (26.2, 33.6)	50.9 (43.4, 58.3)	8.8 (6.8, 11.3)	38.5 (26.3, 52.2)	18.9 (16.8, 21.3)	48.0 (41.5, 54.5)
Marital status						
Single/divorced/widowed	41.7 (37.8, 45.7)	53.5 (47.2, 59.7)	22.9 (19.8, 26.3)	48.7 (43.7, 53.7)	32.0 (29.5, 34.7)	48.7 (43.7, 53.7)
Married/cohabiting	58.3 (54.3, 62.2)	43.4 (38.2, 48.8)	77.1 (73.7, 80.0)	44.0 (40.6, 47.4)	68.0 (65.3, 70.5)	44.0 (410, 47.4)
Employment status						
No	32.0 (28.3, 35.9)	60.1 (52.7, 66.9)	27.9 (24.5, 31.6)	63.8 (56.0, 70.8)	29.9 (37.4, 32.5)	61.2 (56.5, 66.8)
Yes	68.0 (64.1, 71.7)	42.9 (38.0, 47.9)	72.1 (68.4, 75.5)	36.8 (32.4, 41.4)	70.1 (67.5, 72.6)	39.6 (36.3, 43.0)
In debt						
No/little	75.1 (71.7, 78.2)	38.1 (33.9, 42.5)	76.5 (73.3, 79.4)	33.0 (29.2, 37.1)	75.8 (73.5, 78.0)	35.4 (32.6, 38.4)
High	24.9 (21.8, 28.3)	55.0 (47.5, 62.4)	23.5 (20.6, 26.7)	58.3 (50.6, 65.6)	24.2 (22.0, 26.5)	56.6 (51.2, 61.9)

*CI* confidence interval

*Typical mental problems* (somatization, generalized anxiety disorder, and major depression) were sourced from (1) in Thailand validated “*Patient Health Questionnaire-9* (PHQ-9)” [22, 23]. (Cronbach’s alpha 0.88), (2) the “*Generalized anxiety disorder* 7-item (GAD-7)” [24] (Cronbach’s alpha 0.92), and (3) The “*Patient Health Questionnaire-15 somatic symptoms (PHQ- 15)”* [25]. (Cronbach’s alpha 0.83).

*Substance use disorders* were assessed with the in Thailand validated “*Ultrarapid Alcohol, Smoking, and Substance Involvement Screening Test* (ASSIST-Lite)” [26]. Cronbach alpha of the ASSIST-Lite in this study was 0.90.

*Quality of Life (Qol)* was assessed with the World Health Organization Quality of Life (WHOQol)-8, consisting of physical domain (2 items), psychological domain (2 items), environmental domain (2 items), and social domain (2 items) [27, 28]. Each item was scored from 1 (worst) to 5 (best), and summed to result in 2-items subscales and 8-items overall WHOQoL, which was then converted into a 0–100 scale, with higher scores indicating better QoL [18] (Cronbach alpha 0.86).

### Data analysis

The descriptive characteristics of the sample, multimorbidity, and QoL were calculated as percentage, means and standard deviation. Analysis of covariance (ANCOVA) was utilized to assess the associations between five measures of QoL (the four QoL domains: Psychological, Physical, Social and Environment, and overall QoL) and various chronic diseases and adjustments were made for age, sex, employment status, marital status, education, economic status, comorbidity, and health care setting. Multivariable logistic regression was utilized to estimate the predictors of multimorbidity, overall and two treatment settings. Independent variables included sociodemographic factors, quality of life, and type of health care setting. Multi-variable linear regression was used for the assessment of the impact of explanatory variables (socio-demographic factors and type of health care setting) on overall QoL in the multimorbidity population, overall and two treatment settings. *p* values < 0.05% were used to indicate statistical significance. The data were analysed using “IBM-SPSS for Windows, version 25 (Chicago, IL, USA)”.

## Results

### Sample and multimorbidity characteristics

Participants included 1409 attendees of two treatment settings (response rate 97%), 723 of primary care and 686 of monk healers. The age of participants was significantly higher in the health centre (Mean 53.3 years) than in the monk healer setting (Mean 47.3 years). The proportion of two or more chronic conditions (multimorbidity) was 40.6%, 42.4% in the monk healer and 38.9% in the primary care setting. Further sociodemographic characteristics by the percentage of multimorbidity (≥ 2 chronic conditions) are described in Table 1.

### Prevalence of chronic conditions and morbidity

From 12 health care providers diagnosed chronic conditions and two interview-based assessed chronic conditions (common mental disorder and substance use disorder), the most common was common mental disorder (25.2%), followed by hypertension (22.8%), high blood cholesterol (18.0%), fatigue disorder (14.4%), diabetes (14.0%), migraine headaches (13.7%), sleeping problem (12.2%), and ulcer (11.0%). In all, 35.8% had no chronic condition, 23.5% had one chronic condition, 15.1% had two, 9.8% had three, and 15.7% had four or more chronic conditions. The prevalence of emphysema or asthma, osteoporosis, cancer, substance use disorders, heart attack or stroke, common mental disorders, sore joints, and sleeping problems was higher among monk healer attendees, while the prevalence of diabetes and hypertension was higher in primary care attendees (see Table 2).

**Table 2:** Prevalence of chronic conditions and morbidity

Variable	Total sample (N = 1409)	Monk healer (n = 686)	Health center (n = 723)	Chi-square
	% (95% CI)	% (95% CI)	% (95% CI)	*p* value
Hypertension	22.8 (20.7, 25.0)	16.0 (13.5, 19.0)	29.2 (26.0, 32.6)	< 0.001
Heart attack or stroke	4.8 (3.8, 6.1)	7.3 (5.6, 9.5)	2.5 (1.6, 3.9)	< 0.001
High blood cholesterol	18.0 (16.1, 20.1)	17.9 (15.2, 21.0)	18.1 (15.5, 21.1)	0.438
Diabetes	14.0 (12.3, 15.9)	10.3 (8.3, 12.9)	17.4 (14.8, 20.4)	< 0.001
Emphysema/asthma	3.8 (2.9, 5.0)	5.1 (3.7, 7.0)	2.6 (1.7, 4.1)	0.006
Sore joints, e.g., arthritis, gout	9.2 (7.8, 10.9)	12.0 (9.7, 14.6)	6.6 (5.0, 8.7)	< 0.001
Osteoporosis	4.1 (3.2, 5.3)	6.7 (5.1, 8.8)	1.7 (0.9, 2.9)	< 0.001
Cancer or a malignancy of any kind	3.5 (2.7, 4.7)	5.7 (4.2, 7.7)	1.5 (0.8, 2.7)	< 0.001
Migraine headaches	13.7 (12.0, 15.6)	15.5 (12.9, 18.4)	12.0 (9.9, 14.6)	0.067
Ulcer (a stomach, duodenal or peptic ulcer)	11.0 (9.5, 12.7)	10.8 (8.7, 13.3)	11.2 (9.1, 13.7)	0.908
Fatigue disorder	14.4 (12.7, 16.3)	13.8 (11.5, 16.6)	14.9 (12.5, 17.7)	0.689
Sleeping problem	12.2 (10.6, 14.0)	14.4 (12.0, 17.3)	10.1 (8.1, 12.5)	0.012
Common mental disorder	25.2 (23.0, 27.6)	29.2 (25.9, 32.8)	21.5 (18.6, 24.7)	< 0.001
Substance use disorder	8.5 (7.0, 10.2)	11.7 (9.3, 14.5)	5.4 (3.9, 7.5)	< 0.001
Pattern of morbidity				
No chronic condition	35.8 (33.3, 38.4)	30.2 (26.8, 33.8)	41.4 (37.6, 44.9)	< 0.001
One chronic condition	23.5 (21.4, 25.9)	27.4 (24.2, 31.0)	19.9 (17.1, 23.0)	
Two chronic conditions	15.1 (13.3, 17.1)	15.0 (12.5, 17.9)	15.2 (13.0, 18.0)	
Three chronic conditions	9.8 (8.3, 11.5)	9.5 (7.5, 12.0)	10.0 (8.0, 12.5)	
Four or more chronic conditions	15.7 (13.9, 17.8)	17.9 (15.1, 21.0)	13.7 (11.4, 16.5)	
Two or more chronic conditions	40.6 (38.0, 43.3)	42.4 (38.7, 46.2)	38.9 (35.3, 42.6)	0.188

*CI* confidence interval

The percentage with the highest comorbidity was common mental disorder (48.9%), followed by hypertension (48.4%), high blood cholesterol (41.1%), fatigue disorder (33.3%), migraine headaches (30.4%), diabetes (27.9%), sleeping problem (27.0%), ulcer (22.6%), sore joints (18.7%), substance use disorder (13.0%), heart attack or stroke (11.6%), emphysema or asthma (8.5%) and cancer (7.2%). The highest mean multimorbidity reported was for osteoporosis (5.48), followed by emphysema or asthma (5.38), heart attack or stroke (5.32), cancer (5.14), fatigue disorder (4.35), sleep problem (4.29), sore joints (4.20), ulcer (4.03), high cholesterol (3.95), migraine headaches (3.84), diabetes (3.65), hypertension (3.39) common mental disorder (3.28), and substance use disorder (2.54).

### Quality of Life in chronic conditions and multimorbidity

In ANCOVA analysis, adjusted for sex, age, employment status, marital status, education, economic status, comorbidity, and health care setting, the poorest overall QoL was found among clients with common mental disorders (58.5 mean score), followed by emphysema or asthma (60.2), sleeping problem (61.5), migraine headaches (62.7), fatigue disorder (63.3), substance use disorder (63.6) and ulcer (64.3). The highest overall QoL score was found among clients with hypertension (67.6 mean score), followed by high blood cholesterol (67.2), and diabetes (66.7). The overall QoL was significantly higher (71.2 mean score) in clients with no chronic conditions, compared to those with four or more chronic conditions (61.8 mean score).

The overall QoL score was 67.5, while the social QoL domain had the highest score (72.1), followed by the physical QoL subdomain (68.9), and the lowest were in the psychological QoL subdomain (64.0) and the environmental QoL sub-domain (64.7). The overall QoL was poorer among monk healer clients (66.5) than primary care patients (68.8).

Psychological QoL scores were the lowest for common mental disorders (53.1) and emphysema or asthma (54.5), fatigue disorder (57.7), and sleeping problems (58.7). Physical QoL was the lowest for common mental disorders (60.6), emphysema or asthma (61.3), sleeping problem (62.1), osteoporosis (63.8), sleeping problems (64.4) and heart attack or stroke (64.6). Social QoL was the lowest for common mental disorders (63.3), sleeping problem (65.9), emphysema or asthma (66.0), migraine headaches (67.5) and substance use disorder (67.7), while environmental QoL was the lowest for common mental disorder (56.5), sleeping problem (59.2), emphysema or asthma (59.0), and migraine headaches (59.4). In patients with multimorbidity (four or more chronic conditions) scores for the four QoL subdomains (psychological, physical, social and environment) and overall QoL significantly decreased (see Table 3).

**Table 3:** Adjusted quality of life (QoL) according to chronic disease and multimorbidity

Variable	QoL: Psychological	QoL: Physical	QoL: Social	QoL: Environmental	Overall QoL
	Mean (95% CI)	Mean (95% CI)	Mean (95% CI)	Mean (95% CI)	Mean (95% CI)
All	64.0 (63.0, 65.1)	68.9 (67.8, 70.0)	72.1 (71.2, 73.1)	64.7 (63.8, 65.7)	67.5 (66.6, 68.3)
Monk healer	62.6 (61.0, 64.1)	69.3 (67.7, 70.9)	72.0 (70.6, 73.4)	62.0 (60.6, 63.4)	66.5 (65.2, 67.7)
Primary care	66.0 (64.9, 67.2)***	69.3 (68.1, 70.5)	72.4 (71.4, 73.5)	67.4 (66.2, 68.5)***	68.8 (67.9, 69.8)**
Hypertension	64.0 (61.9, 66.2)	68.5 (66.3, 70.8)	72.5 (70.5, 74.5)	65.8 (63.8, 67.8)	67.6 (66.0, 69.5)
Heart attack or stroke	62.2 (57.9, 66.5)	64.6 (60.2, 68.9)*	69.4 (67.3, 71.2)	65.1 (61.0, 69.3)	65.9 (62.3, 69.4)
High blood cholesterol	63.4 (61.1, 65.7)	67.8 (65.3, 70.2)	72.8 (70.6, 75.0)	64.6 (62.4, 66.8)	67.2 (65.3, 69.1)
Diabetes	62.3 (59.6, 64.9)	68.3 (65.5, 71.2)	71.9 (68.4, 74.4)	64.0 (61.4, 66.6)	66.7 (64.5, 68.9)
Emphysema/asthma	54.5 (49.6, 59.4)***	61.3 (56.1, 66.6)**	66.0 (61.3, 70.7)**	59.0 (54.3, 63.7)*	60.2 (56.2, 64.3)***
Sore joints, e.g., arthritis, gout	61.0 (57.9, 64.2)*	67.9 (64.6, 71.3)	71.5 (68.6, 74.5)	61.3 (58.3, 64.3)*	65.5 (62.9, 68.1)
Osteoporosis	60.8 (56.0, 65.5)	63.8 (58.7, 68.8)*	71.0 (66.5, 75.5)	62.6 (58.1, 67.1)	64.5 (60.6, 68.4)
Cancer or a malignancy of any kind	59.5 (55.6, 65.6)*	66.9 (61.6, 72.3)	70.6 (65.8, 75.3)	65.6 (60.8, 70.4)	66.2 (62.1, 70.3)
Migraine headaches	59.5 (56.9, 62.1)***	64.4 (61.7, 67.2)***	67.5 (65.1, 69.9)***	59.4 (57.0, 61.9)***	62.7 (60.6, 64.9)***
Ulcer (a stomach, duodenal or peptic ulcer)	60.1 (57.2, 63.0)**	66.3 (63.2, 69.5)	69.6 (66.8, 72.4)	61.3 (58.5, 64.1)**	64.3 (61.9, 66.7)**
Fatigue disorder	57.7 (55.1, 60.2)***	65.6 (62.9, 68.3)**	68.2 (65.8, 70.7)***	61.8 (59.3, 64.2)**	63.3 (61.2, 65.4)***
Sleeping problem	58.7 (55.7, 61.6)***	62.1 (59.0, 65.2)***	65.9 (63.1, 68.7)***	59.2 (56.4, 62.0)***	61.5 (59.1, 63.9)***
Common mental disorder	53.1 (51.2, 55.0)***	60.6 (57.9, 62.8)***	63.3 (61.4, 65.2)***	56.5 (54.6, 58.3)***	58.5 (56.9, 60.1)***
Substance use disorder	59.3 (55.2, 63.4)*	66.5 (63.4, 69.6)	67.7 (63.8, 71.5)*	60.3 (56.5, 64.2)*	63.6 (60.3, 67.0)*
Pattern of morbidity/multi-morbidity					
No chronic condition	68.2 (66.3, 70.1)	72.8 (70.7, 74.8)	74.8 (72.9, 76.6)	69.0 (67.2, 70.6)	71.2 (69.6, 72.7)
One chronic condition	65.9 (63.8, 68.0)	70.4 (68.1, 72.7)	74.9 (72.9, 77.0)	65.7 (63.7, 67.7)	69.2 (67.5, 71.0)
Two or more chronic conditions	59.6 (58.1, 61.2)	65.3 (63.6, 67.0)	68.7 (67.2, 70.2)	61.1 (59.6, 62.6)	63.7 (62.4, 65.0)
Two chronic conditions	63.3 (60.8, 65.9)	66.1 (63.4, 68.9)	70.6 (68.2, 73.1)	62.4 (60.0, 64.8)	65.7 (63.6, 67.8)
Three chronic conditions	59.5 (56.4, 62.6)	66.0 (62.8, 69.4)	69.5 (66.5, 72.5)	59.8 (56.8, 62.8)	63.9 (61.3, 66.5)
Four or more chronic conditions	56.2 (53.7, 58.6)***	63.9 (61.3, 66.6)***	66.4 (64.1, 68.8)***	60.5 (58.2, 62.9)***	61.8 (59.7, 63.8)***

***p > 0.001; **p < 0.01; *p < 0.05; Adjusted for age, sex, employment status, marital status, education, economic status, comorbidity, and health care setting

### Associations with multimorbidity

In adjusted logistical regression analysis, being a monk healer attendee (Adjusted Odds Ratio-AOR: 1.36, 95% Confidence Interval-CI 1.03–1.78, *p* < 0.05), older age (55–93 years) (AOR: 2.62, 95% CI 1.77–3.86, *p* < 0.001), and high debt (AOR: 1.45, 95% CI 1.09–1.93, *p* < 0.05) were positively, and being employed (AOR: 0.54, 95% CI 0.41–0.73, *p* < 0.001) and better overall quality of life (AOR: 0.96, 95% CI 0.95–0.97, *p* < 0.001) were negatively associated with multimorbidity, overall, for the monk healer and primary care setting. In addition, having post-secondary education was positively associated with multimorbidity in the monk healer setting, and having secondary education (compared to primary or less education) was negatively associated with multimorbidity in the primary care setting (see Table 4).

**Table 4:** Logistic regression model examining determinants of multimorbidity (≥ 2 chronic conditions)

Variable	Monk healer	Primary care	All
	AOR (95% CI)	AOR (95% CI)	AOR (95% CI)
Treatment setting	–	–	
Health centre			1 (Reference)
Monk healer			1.36 (1.03, 1.78)*
Age group			
19–39	1 (Reference)	1 (Reference)	1 (Reference)
40–54	1.03 (0.67, 1.59)	2.14 (1.12, 4.09)*	1.31 (0.93, 1.86)
55–93	2.18 (1.27, 3.75)**	3.90 (2.04, 7.44)***	2.62 (1.77, 3.86)***
Sex			
Female	1 (Reference)	1 (Reference)	1 (Reference)
Male	0.90 (0.58, 1.39)	1.16 (0.77, 1.76)	1.08 (0.80, 1.44)
Formal education			
Primary or less	1 (Reference)	1 (Reference)	1 (Reference)
Secondary	1.20 (0.74, 1.95)	0.47 (0.29, 0.75)**	0.72 (0.52, 1.00)
Post-secondary	1.68 (1.03, 2.76)*	0.83 (0.41, 1.67)	1.27 (0.87, 1.86)
Marital status			
Single/divorced/widowed	1 (Reference)	1 (Reference)	1 (Reference)
Married/cohabiting	0.72 (0.49, 1.05)	1.14 (0.71, 1.83)	0.85 (0.63, 1.13)
Employment status			
No	1 (Reference)	1 (Reference)	1 (Reference)
Yes	0.63 (0.42, 0.94)*	0.47 (0.31, 0.72)***	0.54 (0.41, 0.73)***
In debt			
No/little	1 (Reference)	1 (Reference)	1 (Reference)
High	1.26 (0.85, 1.89)	1.77 (1.16, 2.72)**	1.45 (1.09, 1.93)*
Overall quality of life	0.96 (0.95, 0.97)***	0.97 (0.96, 0.99)***	0.96 (0.95, 0.97)***

*AOR* adjusted odds ratio, *CI* confidence interval

***p > 0.001; **p < 0.01; *p < 0.05

### Associations with quality of life in multimorbidity

In adjusted linear regression analyses, primary health care attenders (Adjusted Coefficient-ACoef.: 6.18, 95% CI 2.93 to 9.42, *p* < 0.001), older age (ACoef.: 0.14, 95% CI 0.02 to 0.27, *p* < 0.05), post-secondary education (ACoef.: 8.77, 95% CI 4.51 to 13.05, *p* < 0.001) and being employed (ACoef.: 3.80, 95% CI 0.69 to 6.90, *p* < 0.05) was associated with higher overall QoL. Female sex, higher formal education, and being married or cohabiting were associated with higher overall QoL among monk healer attenders, and having post-secondary education and being employed were associated with better overall QoL among primary care attenders (see Table 5).

**Table 5:** Multivariable linear regression model for factors associated with overall QoL in multimorbidity population in the monk healer setting, primary care setting and overall

Variable	Monk healer	Primary care	All
	Adjusted Coef (95% CI)	Adjusted Coef (95% CI)	Adjusted Coef (95% CI)
Health care setting	–	–	
Monk healer			Reference
Health centre			6.18 (2.93 to 9.42)***
Age in years	0.17 (− 0.03 to 0.37)	0.03 (− 0.13 to 0.19)	0.14 (0.02 to 0.27)*
Sex			
Female	Reference	Reference	Reference
Male	− 7.63 (− 13.24 to − 2.02)**	2.38 (− 1.73 to 6.50)	− 2.15 (− 5.59 to 1.28)
Formal education			
Primary or less	Reference	Reference	Reference
Secondary	1.31 (3.90 to 7.70)**	1.63 (− 3.90 to 7.15)	1.32 (− 2.93 to 5.55)
Post-secondary	8.25 (1.43 to 14.29)**	10.77 (3.99 to 17.65)**	8.77 (4.51 to 13.05)***
Marital status			
Single/divorced/widowed	Reference	Reference	Reference
Married/cohabiting	5.52 (0.85 to 10.19)*	− 1.99 (− 6.41 to 2.44)	2.47 (− 7.77 to 5.71)
Employment status			
No	Reference	Reference	Reference
Yes	2.50 (− 2.30 to 7.30)	5.25 (1.43 9.07)**	3.80 (0.69 to 6.90)*
In debt			
No/little	Reference	Reference	Reference
High	1.73 (− 3.14 to 6.60)	− 3.73 (− 7.52 0.05)	− 0.88 (− 3.98 to 2.23)

*Coef* Coefficient

***p > 0.001; **p < 0.01; *p < 0.05

## Discussion

This is the first study assessing chronic conditions, multimorbidity, and QoL in patients attending two different treatment settings in Asia. The study found a high prevalence of multimorbidity (40.6%) (42.4% in the monk healer and 38.9% in the primary care setting), which was higher than in primary care in India (28.3% Odisha state, 16.2% in Kerala) [2, 3]. The prevalence of multimorbidity was higher in monk healers than primary care attendees. This result is in line with several studies [4–8] showing that patients with multimorbidity are more likely to utilize traditional and complementary medicine (TCM) than patients without multimorbidity. In this study, clients attending monk healers had more likely mental and substance use disorders than those attending primary care. Patients with coexisting physical and mental disorders have higher functional disabilities and poor quality of life compared to those with physical conditions only, and are therefore more likely to attend monk healers than primary care to improve their functional status, which was not successfully treated in primary care [8]. The lower prevalence of multimorbidity in primary care may be related to lower availability of specialists and supporting services [2], primary care health centres in Thailand provide preventive and basic acute care, mainly provided by professional nurses. Patients with higher education seem to more likely consult monk healers and secondary care (district hospitals) directly. Consistent with a previous review [29], this study found that the highest proportion of comorbidity was found for common mental disorders (48.9%) and hypertension (48.4%). Somewhat similar to a previous study [29], this study found that the highest mean multimorbidity was among patients with emphysema or asthma (5.38), heart attack or stroke (5.32), and cancer (5.14) and to a lower extent for hypertension (3.39).

The study found that the overall QoL was poorer among monk healer clients (66.5) than primary care patients (68.8), but was a little higher than in primary care patients in Brazil (65.2) [21]. In agreement with previous studies, the poorest overall QoL was found among clients with common mental disorders (58.5 mean score), emphysema or asthma (60.2), sleeping problems (61.5), and migraine headaches (62.7), compared to many other chronic diseases, probably due to their more symptomatic presentation [19, 30]. On the other hand, patients reporting more asymptomatic or less disabling conditions, such as hypertension and high blood cholesterol, had better QoL scores, as also found in previous studies [20]. The finding that heart attack or stroke, cancer, and arthritis were not significantly associated with lower overall QoL was unexpected, since previous studies found such associations [18]. Furthermore, the study confirmed that different chronic diseases affected specific domains of QoL differently, as also found previously [18, 19]. Having a common mental disorder or a sleep problem was not only impacting negatively on psychological QoL but also on other QoL subdomains, such as physical, social, and/or environmental, which may have the implication of managing mental and physical problems concurrently [19].

Consistent with some studies [31–33], this study found that older age and lower socioeconomic status (in debt and not employed) was associated with multimorbidity. Moreover, this study confirms findings from a previous review on the existence of a negative association between QoL and multimorbidity [16]. The possible association between QoL and multimorbidity may have implications for health care management in the different health care settings in Thailand [34], especially because being a client of a monk healer was associated with multimorbidity. Unlike some previous studies [31] that found a preponderance of multimorbidity among women, this study did not find any sex differences in the prevalence of multimorbidity.

Consistent with some previous studies [18, 30, 35, 36], this study found that sociodemographic variables (older age, better education, and being employed) were associated with higher QoL scores. Higher education and being employed may be associated with higher awareness of a healthy lifestyle and easier access to health services, which in turn may lead to improved health as well as QoL [37]. Among clients visiting a monk healer, being female and married or cohabiting were associated with better QoL. Being married or cohabiting has a vital role in social support, as opposed to living alone, and may facilitate QoL [37]. To address multimorbidity and improve QoL, a person-centred approach has been proposed by tackling the health issues of a particular patient in a combined fashion [38]. In this line, it would be important to coordinate within the network of healthcare providers from primary care and monk healer settings of care to ensure continuity of care for the patient [38].

This study had several limitations because it was cross-sectional and information was collected by self-report. The study was conducted with patients from conveniently selected monk healer and primary health facilities, who compared to specialist care tended to have probably milder or more stable conditions. Some of the included chronic diseases such as cancer and osteoporosis in the study had small subgroup sample sizes, which limited the detection of associations. The study did not assess the perceived severity of the chronic conditions, which could have an impact on QoL, and should be assessed in future studies.

## Conclusion

Multimorbidity was higher among clients attending monk healers than those attending primary care facilities and QoL was poorer among clients seeking care from monk healers than those attending primary care. The study found a high prevalence of multimorbidity (40.6%) (42.4% in the monk healer and 38.9% in the primary care setting). Older age, having high debt, and being a client of a monk healer increased and being employed and overall QoL decreased the odds of multimorbidity. Being a primary health care attender, older age, post-secondary education and employment was associated with better overall QoL. Determinants of multimorbidity and QoL in two different treatment settings provide information to improve the management of chronic conditions.

## Data Availability

The data for the current study will not be shared publicly as participants were informed at the time of providing consent that only researchers involved in the project would have access to the information they provided.

## Abbreviations

ANCOVA: Analysis of Covariance
ASSIST-Lite: Ultrarapid Alcohol, Smoking, and Substance Involvement Screening Test
COPD: Chronic obstructive pulmonary disease
CVD: Cardiovascular disease
GAD: Generalized anxiety disorder
PHQ: Patient Health Questionnaire
QoL: Quality of Life
TCM: Traditional and complementary medicine
THP: Traditional health practitioner
